# A Case report: Synovial sarcoma of the mediastinum in an 18-year-old teenager

**DOI:** 10.3389/fonc.2024.1288213

**Published:** 2024-02-16

**Authors:** Yan Liu, Manman Cui, Xiuzhi Zhou, Duchang Zhai, Mingyu Qin, Guohua Fan, Wu Cai

**Affiliations:** ^1^ Department of Radiology, The Second Affiliated Hospital of Soochow University, Suzhou, China; ^2^ Suzhou Medical College, Soochow University, Suzhou, China

**Keywords:** synovial sarcoma, mediastinum, mesenchymal cell, chest imaging, oncology

## Abstract

Synovial sarcomas (SSs) are a rare group of malignant tumors originating from pluripotential mesenchymal cells, which commonly occur as the primary tumor in the soft tissues near the articular surface, tendons, and articular synovium. Herein, we report a rare case of mediastinal SS in an 18-year-old teenager who initially presented with cough as the primary symptom. In this case, plain chest CT and contrast-enhanced CT clearly revealed the lesion presenting as a round-like and uneven density mass in the mediastinum with heterogeneous enhancement, which compressed the trachea and invaded the adjacent vessels. Based on the results of immunohistochemistry and fluorescence *in situ* hybridization (FISH), combined with the differential diagnosis with other types of tumors in the mediastinum on imaging, we were able to diagnose the tumor as an SS located in the mediastinum. Subsequent resection of the lesion coupled with chemotherapy and immunotherapy led to an improvement in the patient’s symptoms.

## Introduction

Synovial sarcoma (SS) is a highly aggressive mesenchymal tumor that rarely appears in the mediastinum. Similar to other mediastinal tumors, patients with mediastinal SS have a younger mean age of onset. The clinical manifestations and imaging features of SS are nonspecific and often present with respiratory-related chest pain, cough, hemoptysis, dyspnea, and a mass of soft-tissue density in the mediastinum. The diagnosis of SS mainly relies on the combination of pathological, immunohistochemical, and molecular genetic analyses. Here, we described a case of an 18-year-old teenager with mediastinal SS who presented with coughing and a heterogeneous mass in the superior mediastinum.

## Case report

**Figure 1:**
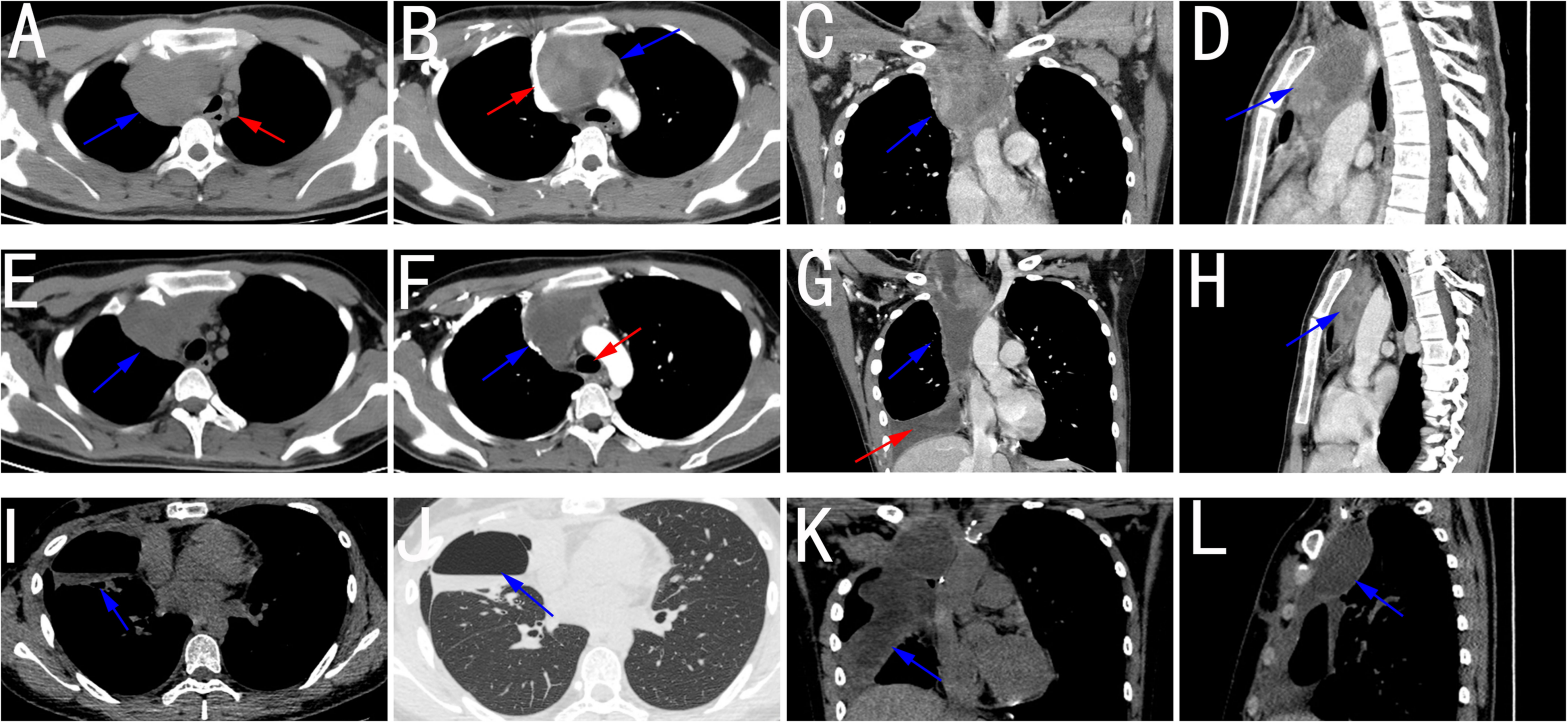
**(A)** Chest plain CT scan showing a mass with an irregularly round shape (blue arrow) that compressed the adjacent structure (red arrow) in the superior mediastinum, including the trachea and vessels. **(B)** Contrast-enhanced chest CT scan showing that the enhancement of the lesion was uneven and obvious (blue arrow), with a compressed and irregularly narrow superior vena cava (red arrow). **(C, D)** Coronal and sagittal reconstruction showing the position of the cystic and solid mass (blue arrow) in the superior mediastinum. **(E, F)** After a four-month course of combined chemotherapy, the chest CT scan and contrast-enhanced chest CT scan showed shrinkage of the mass and decreased compression of surrounding structures, such as the trachea (red arrow). **(G, H)** Coronal and sagittal reconstruction also showing a reduction in the mass volume and a decrease in the solid component (blue arrow), accompanied by pleural effusion (red arrow). **(I, J)** CT scan two months after surgery showing hydropneumothorax in the thoracic cavity (blue arrow). **(K, L)** CT scan two months after surgery showing encapsulating cystic fluid accumulation near the surgical area (blue arrow).

An 18-year-old teenager was admitted to the hospital due to an unrelieved cough. The patient reported a one-week history of coughing without sputum, as well as right chest pain that worsened after severe coughing and exercise. A plain chest CT at the outpatient clinic revealed a round soft tissue mass that compressed the trachea toward the left, leading to a diagnosis of neoplasm in the right neck extending to the mediastinum (**Figure 1A**). The patient was then admitted for further examination and treatment. Laboratory test results showed no obvious abnormalities. A contrast-enhanced chest CT scan demonstrated a cystic and solid soft tissue mass measuring 7.8×5.2×8.3 cm located in the right lower neck to the anterior superior mediastinum. The mass had clear borders and uneven density with patchy areas of low density, which is characterized by marked and heterogeneous enhancement (**Figures 1B, D**). Additionally, adjacent blood vessels exhibited indistinct boundaries due to invasion, particularly the irregular narrowing of the invaded superior vena cava (**Figures 1B, C**). No significant enlarged lymph nodes were found in the bilateral hilar area and mediastinum. Based on the aforementioned imaging results, the imaging diagnosis was suspected to be a germ cell tumor (seminoma). Laboratory examination (including routine blood and coagulation function examinations) and physical examination of the patient before surgery showed no obvious abnormalities. In view of the large size of the tumor and the high risk of complete surgical resection, an intraoperative pathological biopsy was performed via right thoracoscopic surgery. After the surgery, the patient was discharged in stable condition.

**Figure 2:**
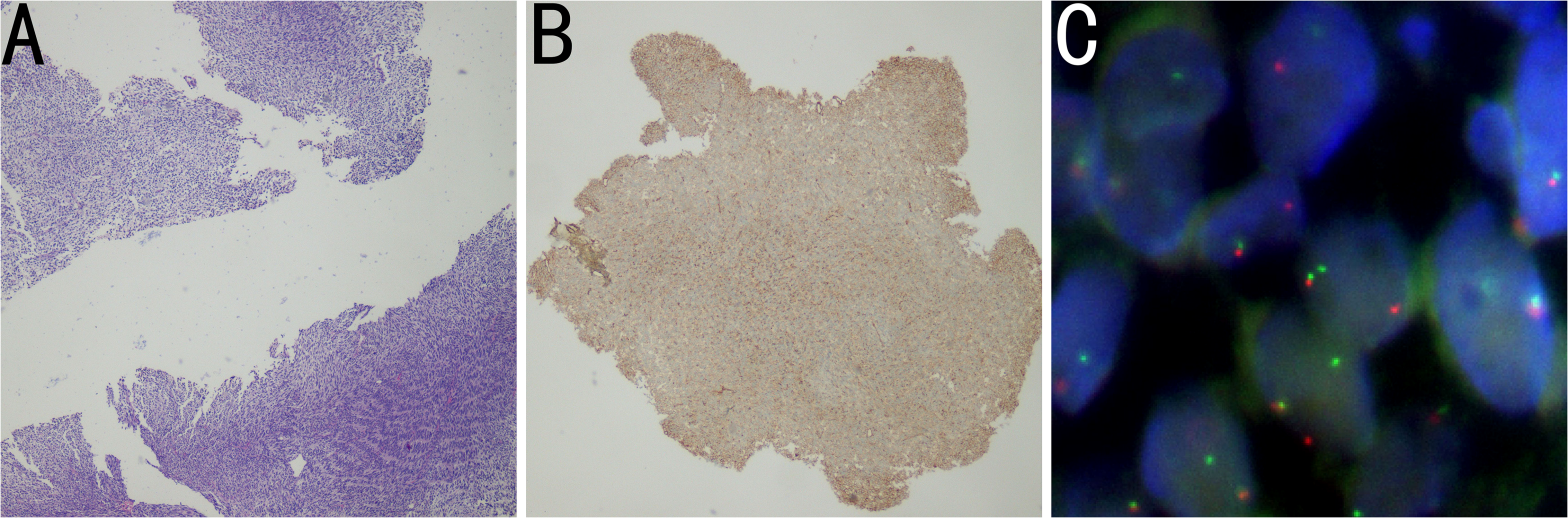
**(A)** Histopathology image showing tumor cells composed of malignant short spindle cells (H&E staining). **(B)** Immunohistochemistry image showing positivity for CD99. **(C)** FISH results showing that the proportion of *SS18* (18Q11) SYT:1R1G1F signal type abnormal cells was 24%, and the *SYT* gene was broken. The FISH results were positive.

Subsequently, the histopathologic examination of the mass indicated a number of malignant short spindle cells (**Figure 2A**), while the immunohistochemistry results showed that the cells were positive for CD99 (**Figure 2B**), Bcl-2 and calponin but negative for AE1/AE3, EMA, NKX2.2, and WT1, while Ki-67 was 40%. Meanwhile, the result of fluorescence *in situ* hybridization (FISH) was positive (**Figure 2C**), which revealed translocation of t (18q 11.2) (*SS18*), and the *SYT* gene was broken. The above results confirmed the diagnosis of mediastinal SS.

Consequently, the patient received the combined therapy of immunotherapy with PD-1 monoclonal antibody and chemotherapy with doxorubicin and ifosfamide, where the dose of PD-1 was 200 mg every three weeks, doxorubicin was 60 mg day one, and isocyclophosphamide was 4 g days 2-4 every three weeks. After five courses of combined therapy, contrast-enhanced CT showed that the mass had shrunk to a size of 7.5×3.2×6.9 cm with the presence of pleural effusion, accompanied by a decrease in its solid component and reduced compression of surrounding structures (**Figures 1E, G, H**). Meanwhile, contrast-enhanced CT imaging still showed uneven and significant enhancement of the mass (**Figures 1F– H**). Complex mediastinal tumor resection was performed when the condition of the tumor was eligible for surgical indications. Postoperative pathology showed that the SS located in the anterior mediastinum and invaded the soft tissue of the chest walls and lung tissue. Most areas of the tumor were necrotic, and some showed fibrous tissue hyperplasia. Tissue cell hyperplasia and small focal multinucleated giant cell reaction were also observed.

Subsequently, a CT scan performed two months after surgery revealed the chest condition post-operation: slight atelectasis of the right lung with hydropneumothorax in the thoracic cavity (**Figures 1I–L**). Hereafter, postoperative adjuvant immunotherapy combined with chemotherapy was continued. To date, the patient has recovered well.

## Discussion

Primary SS does not originate from synovium, but is named because the tumor resembles synovial tissue when viewed at the light microscope (1). In, 2002, the World Health Organization defined SS as a mesenchymal tumor originating from pluripotent mesenchymal cells that can differentiate into epithelial cells. Primary SS accounting for 5-10% of all soft tissue tumors, and according to Ouji et al. primary SS of the mediastinum is rare, which usually occurs in adolescents to young adults (15-40 years old) and predominantly in males (2). Engelhard et al. deemed that mediastinal SS accounts for 10.4% of all mediastinal sarcomas and 0.1% of all sarcomatoid tumors (3). SS occurs mainly in the soft tissues of the extremities, especially the lower limbs, but also in rare sites such as the pericardium, lungs, abdominal wall, genitourinary tract and head and neck, while occurrence in the mediastinum is extremely rare (4). Thus, the case we presented can help us further understand this malignancy. It has a large size, with an average of 7.5 cm, and can compress the surrounding tissues, even leading to displacement; the tumor can also invade adjacent tissues and organs, including the heart, lung, blood vessels, etc (5). Clinical symptoms associated with compression by the tumors are usually nonspecific. In the present case, the cough and the chest pain after severe coughing and exercise might have been caused by a tumor in the superior mediastinum compressing the trachea. Currently, thoracic SS occurs in three main locations: lung parenchyma, pleura and mediastinum, with those occurring in the mediastinum being the rarest. Intrathoracic SS tends to occur in males, has a larger size and extensive tumor necrosis, and is also more aggressive, with a high tendency to invade the neurovascular system, presenting as the SYT-SSX1 variant frequently. SS in the mediastinum, on the other hand, is commonly well defined, has a rounded or lobulated contour, is associated with pleural effusion or pleural infiltration, and mediastinal lymph node involvement is rare.

From a pathological perspective, SS has a biphasic pattern and consists of sheets of spindle cells and sharply segregated epithelial cells forming gland-like areas in varying proportions (6). Accordingly, SS can be classified into four types: monophasic epithelial cell type, monophasic spindle cell type, biphasic type, and hypo-differentiated type. It has been recently proposed that vimentin, cytokeratin, and epithelial cell membrane antigen (EMA) coupled with negative CD34 are the most effective and sensitive protein biomarkers for the diagnosis of SS (6). In addition, positive Bcl-2 can also be used as another biological marker for auxiliary diagnosis. Bcl-2 appears important to distinguish between SS and solitary fibroma. However, the latter also shows CD34 positivity, which is not seen in SS (7). Moreover, it is uniquely distinguished by chromosomal translocation t (X;18) (p11; q11), which replaces the 8 terminal C-amino acids of the *SYT* gene of chromosome 18 with the 78 terminal Camino acids of *SSX1* and *2* of the X chromosome (8). In the current case, we verified the diagnosis of SS because the cells were positive for CD99 and Bcl-2 but negative for EMA, and the FISH result for t(X;18) was positive.

Of note, given that the component of the mediastinum is complex, containing numerous pluripotent mesenchymal cells and various anatomical structures, the differential diagnosis regarding mediastinal neoplasms must be taken into consideration, including malignant thymoma, malignant mesothelioma, teratoma, seminoma, and solitary fibroma. Generally, due to calcification, hemorrhage and necrosis, imaging of mediastinal SS shows a heterogeneous mass with heterogeneous enhancement (9). In magnetic resonance imaging (MRI), the mass shows moderate or slightly low signals on T1-weighted imaging and mixed signals dominated by high signals on T2-weighted imaging. Some scholars describe this mixed high, medium and low signal on T2-weighted imaging as the “Triple Signal “ sign, but not all SS can have the “triple signal sign”, which has been reported to occur in 43%-46.7% of SS in the literature. In terms of thymoma, which often couples with myasthenia gravis, the imaging findings are similar with those of SS; however, calcification is rare, and the tumor is more prone to necrosis and cystic changes. At the same time, p63 is positive in thymoma. Malignant mesothelioma, often with a history of asbestos contact, extensive nodules or irregular thickening of the pleural membrane, can be accompanied by rib destruction and pleural effusion. In addition, teratomas, the most common anterior mediastinal germ cell tumors, have a mixed density of fat, calcification, bone, and soft tissue. When a solid mass with uniform soft tissue density but no fat or calcification occurs in the mediastinum, which is accompanied by increased β-HCG, seminoma should be suspected. Finally, solitary fibrous tumors show tortuous vascular shadows around and within the tumor on contrast-enhanced CT and have the characteristics of delayed enhancement owing to rich fiber components.

Primary SS is highly aggressive with a poor prognosis. Factors affecting prognosis are tumor older age, male, size greater than 5 cm and the presence of vascular, nerval or skeletal metastases. Hypo-differentiated SS (highly mitotic, 10 or more mitoses per 10 microscopic fields) has a poor prognosis. Another poor prognosis is primary SS with necrosis and tumors located in the trunk. Again, SS with the SSXI gene translocation has a worse prognosis than those with the SSX2 gene translocation. The 5-year life expectancy for primary SS in the thorax is 30%, and 50% for primary SS of soft tissue (10, 11). At present, complete surgical resection of the tumor is considered the paramount treatment (12). Studies have indicated that patients who are able to undergo complete surgical resection experience better survival benefits than those with unresectable tumors or positive surgical margins (13). The standard of initial treatment for large tumors, positive margins, lymph node involvement, high-grade, and advanced or metastatic SS is combination chemotherapy with an anthracycline and ifosfamide (14). For larger tumors (>5 cm) or SS requiring preservation of the neurovascular structures or bone, studies have suggested neoadjuvant or adjuvant radiotherapy. At present, targeted medical therapy has been gradually applied to the treatment of SS. Pazopanib, a receptor tyrosine kinase inhibitor, has been approved for clinical use (15). However, preoperative chemotherapy can lead to an increased risk of postoperative wound complications, and postoperative radiotherapy can lead to the development of fibrosis and joint stiffness (16). Therefore, the particularly treatment should be determined by a multidisciplinary team based on the patient’s age, performance status, complications, tumor location, and histological subtype. Therefore, in the current case, the mass shrank after five courses of combined therapy and was eligible for surgical intervention. Meanwhile, the patient was in good overall condition after complete surgical resection and combined chemotherapy. No further radiotherapy was used, as the patient had positive surgical margins and had been treated with chemotherapy combined with immunotherapy to further prevent local and systemic recurrence.

## Conclusion

In summary, primary SS which occurs in the mediastinum characterized by a large heterogeneous lump in the mediastinum as the single initial imaging feature is exceedingly rare, and this may be a factor contributing to misdiagnosis and delayed diagnosis. Considering the low prevalence of SS in the chest, the absence of specific clinical manifestations, the lack of uniform and effective treatment options, along with its poor prognosis, it is essential to perform immunohistochemistry and molecular cytogenetic examination (such as FISH) based on imaging differential diagnosis. Once diagnosed, comprehensive treatment based on surgical resection can be adopted according to the actual situation of the patient, which can further improve the median survival time of the patient.

## Data availability statement

The original contributions presented in the study are included in the article/supplementary material. Further inquiries can be directed to the corresponding author/s.

## Ethics statement

Ethical approval was not required for the studies involving humans because Article only collected the related in the disease process in patients with clinical data, not to implement any experimental operation on the patients. The studies were conducted in accordance with the local legislation and institutional requirements. The participants provided their written informed consent to participate in this study. Written informed consent was obtained from the individual(s) for the publication of any potentially identifiable images or data included in this article.

## Author contributions

YL: Writing – original draft, Writing – review & editing. MC: Writing – original draft. XZ: Writing – review & editing. DZ: Writing – review & editing. MQ: Writing – review & editing. GF: Writing – review & editing. WC: Writing – original draft, Writing – review & editing.
